# Why do women live longer than men, but spend more time in poor health? A decomposition analysis of the gender gap in unhealthy life years across Europe

**DOI:** 10.1007/s10654-025-01346-2

**Published:** 2026-01-12

**Authors:** Magdalena Muszynska-Spielauer, Paola Di Giulio, Yuka Minagawa, Vanessa Di Lego, Marc Luy

**Affiliations:** 1 Vienna Institute of Demography, Austrian Academy of Sciences, Vienna, Austria; 2 Faculty of Liberal Arts, Sophia University, Tokyo, Japan; 3 Demography Department, Cedeplar, Universidade Federal de Minas Gerais, Belo Horizonte, Minas Gerais Brazil

**Keywords:** Gender health-mortality paradox, Unhealthy life years, Longevity hypothesis, mortality and morbidity decomposition

## Abstract

This study tests the “longevity hypothesis,” which posits that women’s greater number of years spent in poor health is primarily a direct consequence of their longer survival. We analyse gender differences in unhealthy life years (ULY) at age 50 across 22 European countries in 2015–2017. ULY was estimated using three approaches—the Sullivan method, the cross-sectional average length of healthy life, and multistate life tables—applied to four health indicators of varying severity: chronic diseases, functional limitations, self-rated health, and disability. Data were drawn from the Human Mortality Database and the Survey of Health, Ageing and Retirement in Europe. We decomposed the gender gap in ULY into a “mortality effect” (ME), reflecting differences in life years lived, and a “health effect” (HE), reflecting differences in morbidity prevalence. Women at age 50 lived more unhealthy years than men across almost all health indicators and countries. In most cases, more than half of the gender gap in ULY was attributable to the ME, indicating that women’s longer survival primarily explains their greater number of years spent in poor health. The HE showed greater variation across indicators and countries. Results were most consistent for chronic diseases and self-rated health, while functional limitations and disability yielded smaller and less consistent differences. Findings support the longevity hypothesis: women’s higher life expectancy is the main driver of their longer lifetime spent in poor health. The variation across health dimensions highlights the importance of distinguishing between them when studying gender inequalities in health.

**Supplementary Information:**

The online version contains supplementary material available at 10.1007/s10654-025-01346-2.

## Introduction

Empirical findings consistently show that women live longer than men [1]. Since the first decade of this century, the survival advantage of women has been documented for every country [2] and it persists even under extreme exogenous conditions such as severe famines and epidemics [3]. Notably, during the Covid-19 pandemic, despite experiencing similar or even higher infection rates, women consistently exhibited lower mortality than men [4].

While women on average live longer than men, this does not mean that they are healthier. Extensive literature reports that women have worse self-rated health [5, 6], spend more years with physical and mental health problems [7–9], have a faster rate of physical function decline [10], are less likely to recover from disability [11], report pain more often [12], and have a greater need for long-term care compared to their male counterparts [13]. These apparent contradictions have led researchers to term the phenomenon the “gender and health paradox” [14], “morbidity-mortality paradox” [15], “paradox of weak but strong women and tough but weak men” [16], or “male-female health-mortality paradox” [17].

While there is compelling evidence of these gendered patterns in health and mortality, some researchers have questioned the existence of this paradox: gender differentials in health are not universal and largely depend on age, health indicator, time, and social context [14, 15, 18–21]. Recently, it has been shown that there are also differences in reporting behavior between women and men which affect gender differences in health expectancy [22, 23]. Also, there is evidence that the differences in the types and severity of health conditions might explain the apparent contradiction of women’s lower mortality and higher morbidity: women suffer more often than men from long-term but nonfatal diseases, whereas men experience more life-threatening conditions with significant mortality consequences, such as heart disease [14, 20, 24–28].

The present paper provides further evidence to support the criticism of interpreting gender differences in health and mortality as a paradox. We demonstrate that it is not contradictory that women’s higher life expectancy is accompanied by a greater number of unhealthy years lived. Drawing on the “longevity hypothesis” by Luy and Minagawa [29], which says that women’s higher number of life years spent in poor health is a direct consequence of their longer survival, this study offers a deeper understanding of how mortality and morbidity jointly explain the seeming contradictory gender differences in morbidity and mortality. We decompose gender differences in unhealthy life years (ULY) at age 50 across 22 countries in Europe, for four different health indicators with different degrees of severity, and three different approaches for the estimation of ULY, in gender differences in mortality (“mortality effect”) versus gender differences in health (“health effect”). According to the longevity hypothesis, gender differences in ULY should be attributable primarily to the mortality effect.

## Data and methods

The calculation of ULY requires data on (i) age-specific mortality rates and (ii) age-specific prevalence of poor health. Data on mortality was taken from period life tables and cohort life tables from the Human Mortality Database [30]. Data on age- and sex-specific prevalence of morbidity for ages 50 and older was taken from the Survey of Health, Ageing and Retirement in Europe (SHARE). We used SHARE Wave 7 data to estimate the prevalence of morbidity in 22 European countries for the Sullivan and HCAL analyses. Multistate life table estimations were based on individual health trajectories between Waves 6 (2015) and 7 (2017) [31, 32]. Sample sizes by country and sex are shown in Table S1 and S2 in Supplementary Material 1.

For the prevalence, we focused on four widely used self-assessed health measures: (i) presence of chronic diseases, (ii) functional limitations, (iii) poor self-rated health (SRH), and (iv) limitations in activities of daily living (ADL). These measures were operationalized following standard SHARE definitions (see Appendix A1 in Supplementary Material 1 for full question wording and coding). Poor health was defined as reporting a chronic disease, any functional limitation, fair or poor SRH, or difficulty performing at least one ADL.

We estimated ULY at age 50 using three complementary approaches (see Appendix A2 in Supplementary Material 1 for details). First, the Sullivan method derives the prevalence of poor health by age from cross-sectional survey data and combines it with life table information on person-years lived [33]. The Sullivan method has the least data requirements. It is simple and widely used, but it was questioned as an appropriate measure of population health in the situation of rapid changes in health transitions or dynamic population health shifts [34, 35]. Second, the HCAL method, an extension of the cross-sectional average length of life (CAL) approach, replaces period person-years with cohort person-years lived, thereby linking health prevalence to cohort mortality histories [36]. The HCAL is based on the understanding that health prevalence observed in a study period results not only from the health conditions during the observation year(s), but also from the health conditions experienced by the cohorts over their complete lifetime from birth until the study period. Third, the multistate life table method is based on estimated transition probabilities between health states, with death as an absorbing state, using longitudinal SHARE data [37]. Multistate models allow for dynamic modeling of health transitions, which gives a more accurate picture of period-specific population health when health conditions change rapidly over time [34, 35]. The three variants of ULY measure different aspects of population health and mortality conditions in a study period. ULY derived with the Sullivan method summarizes age-specific mortality rates and prevalence of the defined state of adverse health condition in the life table population of a study period. ULY based on the HCAL approach measures the average number of unhealthy years lived in a given period over all cohorts alive in that period. ULY derived with the multistate life tables summarizes the outcome of the health processes that occur in the study population during a given period.

To address the research question on how gender differences in health and longevity shape the gap in ULY, we apply the decomposition method of Nusselder and Looman [38]. The decomposition splits the total gender gap in ULY into two parts: “mortality effect” (ME) and “health effect” (HE). The ME captures the gap due to differences in the number of years lived at each age, which result from differential survival at the study age and younger.

## Results

**Fig. 1:**
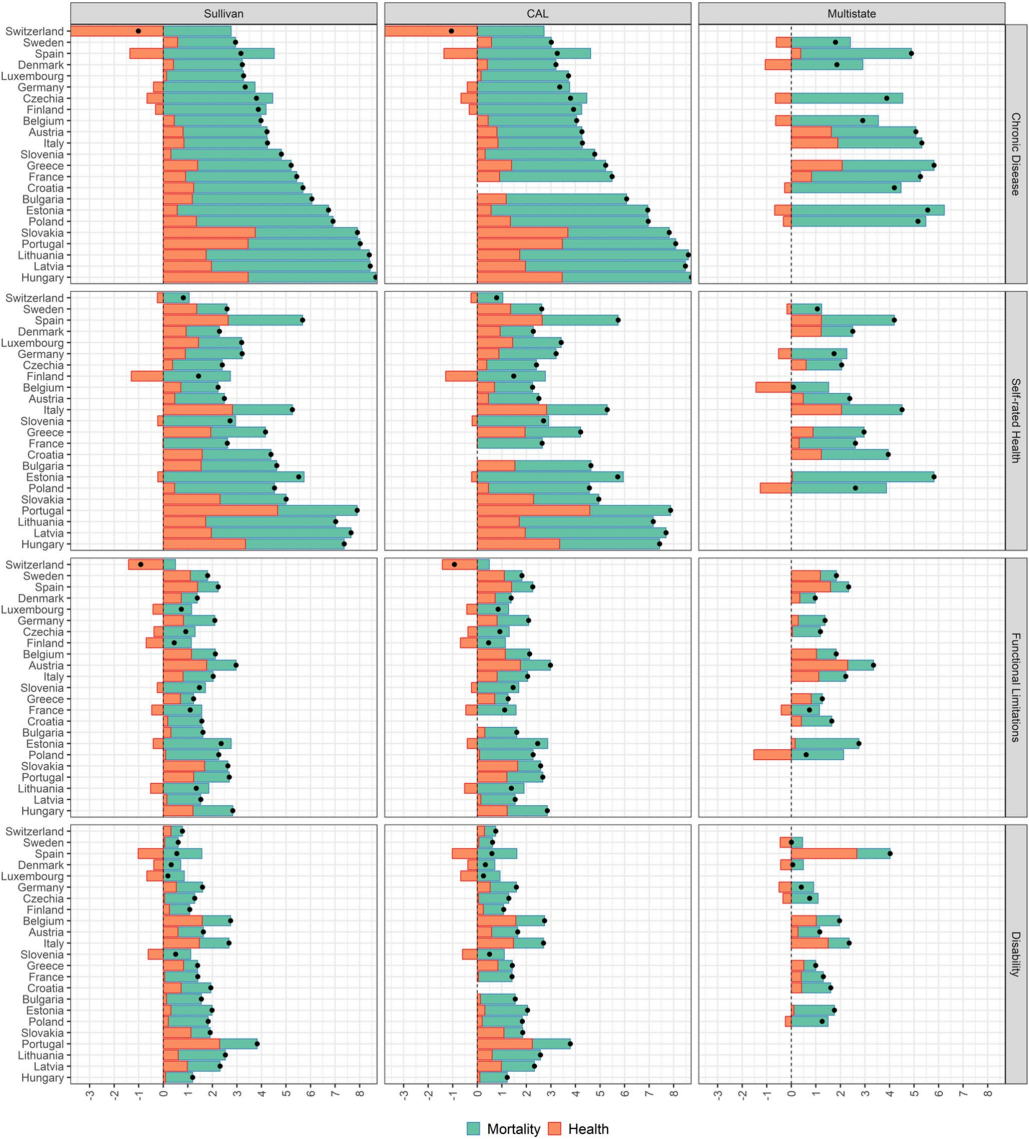
Gender gap in unhealthy life years at age 50 (female minus male) in four health dimensions and three estimation methods. Contribution of health and mortality effects to the gender gap. The countries are ordered by the gender gap in ULY for chronic diseases derived according to the Sullivan method

The results of the decomposition of gender differences (women minus men) in ULY at age 50 into the effects caused by gender differences in mortality (ME) and prevalence of poor health (HE) for 22 European countries are summarized in Fig. 1 (see also Tables S3–S6 in Supplementary Material 1). The countries are ordered by the gender gap in ULY for chronic diseases derived according to the Sullivan method. The bars illustrate the two effects, with the ME being illustrated in green and the HE in red colors. ME and HE sum up to the total gender gap in ULY, which is illustrated by black dots.

A look at these dots shows that the gender gap in ULY is consistently positive, confirming that women on average spend more life years in poor health compared to men. There are only two exceptions: Switzerland, which has a negative gap when estimating ULY for chronic disease with the Sullivan and the HCAL approaches, and Sweden with a zero gap in gender differences in ULY for disability when estimated with the multistate approach. These exceptions are probably at least partly related to the fact that life expectancy for men in Switzerland and Sweden is exceptionally high, resulting in the smallest gender gap in years lived and ULY across the study countries (see Tables S7–S9 in Supplementary Material 1).

Because women live on average longer than men, the ME on the total gender gap in ULY is positive across all the four health indicators and all the three analytical approaches for all countries. The HE of the gender gap in ULY shows more variation. Most countries have also positive HE values, meaning that in these populations the gender gap in ULY caused by the ME is further increased by the higher prevalence of poor health among women. However, there are some cases with negative HE, i.e., countries in which men are characterized by a higher prevalence of poor health compared to women, what narrows the gender gap in ULY caused by the ME. Nonetheless, in all these cases, the overall gender gap in ULY remains positive because the negative HE is counterbalanced by a larger positive ME.

**Fig. 2:**
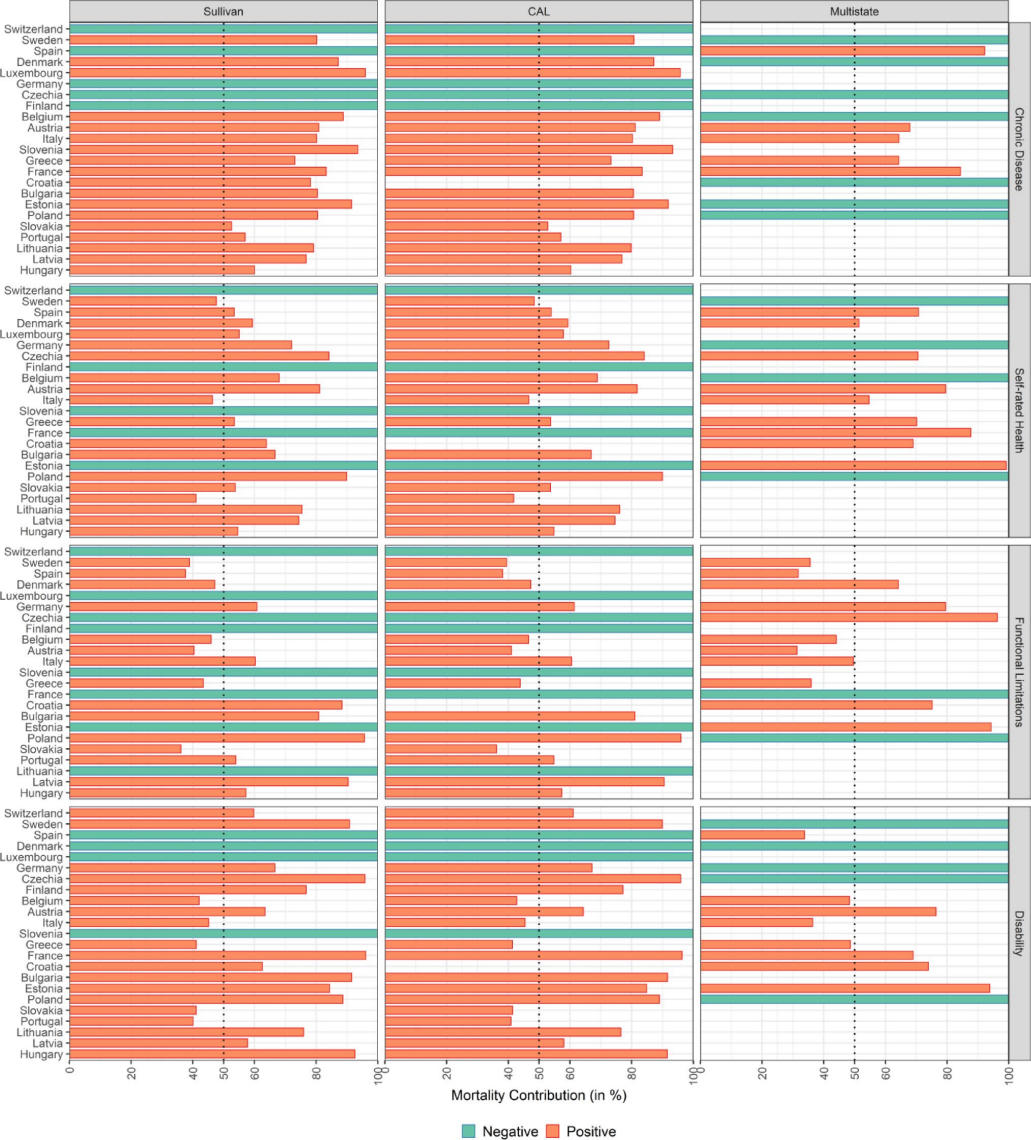
Contribution of mortality to the gender gap in unhealthy life years at age 50 (female minus male) in four health dimensions and three estimation methods, in percent values. Negative contribution means that either the gender gap was positive or the contribution of the health effect was negative. In these cases, we plot contribution of mortality effect as 100%. Countries ordered by the gender gap in ULY for chronic diseases derived according to the Sullivan method. Data Source: SHARE Waves 6 and 7 and Human Mortality Database (2024).

For testing the longevity hypothesis, the most important finding is that in the majority of countries, more than half of the gender gap in ULY can be attributed to the ME. This means that in most countries, the ME has a larger effect on the gender gap in ULY than the HE. This is also illustrated in Fig. 2, which shows the relative contributions of the ME to gender gap in ULY. The only exceptions are Austria, Belgium, Greece, Italy, Spain, and Sweden, where the mortality effect (ME) contributes less than 50% to the gender gap in ULY, albeit in different health dimensions.

Figures 1 and 2 reveal that the relative contribution of the two effects varies with the health dimension for which the gender in ULY is estimated. For chronic diseases and SRH, the ME is generally large and sensibly greater than the HE. As for functional limitations and disability, the contributions of the ME are distinctly smaller. Nevertheless, also when health is measured according to these two dimensions, the ME exceeds the HE by at least one analytical method in all countries except Switzerland.

## Discussion

This study used the measure of ULY at age 50 and revisited the apparent contradiction of women’s longer life and more years spent in poor health, which has often been labelled a paradox. We found that at age 50 women spent more life years in poor health than men, and that these differences in ULY were primarily attributable to the mortality effect (ME), i.e., differences in women’s and men’s total number of life years. While the contribution of the ME to the total gender gap in ULY was consistently positive, the health effect (HE) was more variable between countries and health indicators, but in almost all cases smaller than the ME. Our findings align with previous decomposition studies showing that women’s longer survival is the dominant contributor to the gender gap in unhealthy life years: In Van Oyen et al. [39], Nusselder et al. [40], and Luy [41], the mortality effect consistently outweighed the health effect, which was smaller and also more variable across countries and health dimensions.

Greater effects of mortality on the total gender gap in ULY imply that women’s higher life expectancy is mainly responsible for their greater number of life years spent in poor health, offering support for the longevity hypothesis. Our results therefore confirm Luy and Minagawa’s [29] conclusion that “women suffer from worse health than men do not in spite of living longer, but because they live longer” (p.17).

Consistent with previous findings [6, 40–42], our study confirms further that the extent of the gender gap in ULY depends strongly on the health dimension analysed. We find that the differences between women and men in the number of life years spent in poor health tends to be large when health is defined according to chronic diseases and SRH, and comparatively small for functional limitations and disability. While the occurrence of this variation by health dimension has been repeatedly noted, the reasons for its variation remain an open question. Luy [41, 43] suggests that the main reason for this variation lies in the severity of the diseases underlying different health dimensions. The number of life years spent in poor health rises with total life years, and this increase in ULY is more pronounced when the mortality risk associated with poor health is lower. In modern societies, gains in life expectancy occur mainly at older ages. As the risk of health problems rises with age, such conditions accumulate and are more common in the oldest age groups—especially those conditions that do not necessarily increase mortality. In other words, because people typically spend more years with less severe than with critical health problems, the additional life years that distinguish populations with different life expectancies, such as women and men, are largely years lived with less severe conditions. These milder problems can more frequently be found in dimensions such as chronic diseases, whereas the causes of functional limitations and disability are generally linked to more serious health problems with a stronger relationship on mortality.

This explanation is consistent with the observation that the populations in our study can be divided roughly into two groups in terms of the extent of gender differences in ULY and its variation by health dimension: those with lower levels of life expectancy for both sexes and bigger gender gap in ULY, exemplified by some of the Eastern European countries (Croatia, Slovenia, France, Bulgaria, Hungary, Poland, Estonia, Latvia and Lithuania), and those with higher life expectancy and smaller gender gap in ULY (all others). In general, the longevity hypothesis is confirmed more clearly and across all health dimensions for the first group of countries. For the remaining countries, Switzerland, Sweden, Austria, Portugal, Spain, Italy, Greece, Slovakia, and Belgium, we observe similar results as for the first group for chronic conditions and SRH, but we find a less consistent picture for disability and functional limitations. Taken together, these results indicate that the larger the gender gap in ULY, the larger the ME. Note that the extent of the gender gap in ULY depends both on differences in life expectancy and on the health dimension considered.

There are some limitations to our study that need to be noted. First, it is important to stress that this study draws on broad health indicators, which may not be transferable to all health characteristics. In particular, many minor or specific illnesses with known gender differences are not reflected in the aggregated measures considered here. Therefore, our results do not rule out the possibility that there are specific diseases which occur more frequently and severely in women than in men.

Second, the decomposition of the gender gap in ULY into ME and HE provides insights into the effects on the total number of life years spent in poor health, but gives no details on the specific origins of the underlying processes, such as gender differences in age-specific mortality rates or transitions between health states. Instead, the applied decomposition isolates the separate contributions of two population-level components: life years lived, as determined by the age-specific mortality rates of the period, and the prevalence of poor health. This distinction is important because ULY is a population-level aggregate outcome measure and not a measure of the processes that determine these outcomes. While these two components are endogenous to each other - differential mortality by health status influences health prevalence, and health prevalence in turn influences total mortality - this endogeneity does not invalidate the decomposition. As long as we recognize this interplay and interpret the results as reflecting the effects of these two aggregate components rather than differences in the underlying processes, the conclusions drawn from such a decomposition remain valid.

Finally, as demonstrated by Muszyńska-Spielauer and Spielauer [44], the SHARE data applied in the study is subject to selective attrition on health and other characteristics that influence health, raising the possibility that our estimates of ULY may be biased. While younger individuals in good health are more likely to leave the predominantly longitudinal study sample, at older age it is individuals in the poor health that are more likely to attrite. Additional to health-selected attrition, as men are more likely to leave the longitudinal samples [45, 46], the differences between women and men in the prevalence of poor health are likely to be biased by attrition.

Conversely, our study also has important strengths. To the best of our knowledge, this is the first study to explore the mechanisms behind the relationship between life expectancy and years spent lived in poor health by gender using such broad cross-national coverage and a wide variety of health indicators and methodological approaches to estimate ULY. These strengths increase the robustness of our results and conclusions, thereby advancing our understanding of the mechanisms underlying the gender gap in ULY in European countries.

## Supplementary Information

Below is the link to the electronic supplementary material.

Supplementary Material 1
