# Supplementary material for: Why do women live longer than men, but spend more time in poor health? A decomposition analysis of the gender gap in unhealthy life years across Europe
Source: Eur J Epidemiol. 2026 Jan 12;41(3):329–36. doi: 10.1007/s10654-025-01346-2 (PMC13222172; doi:10.1007/s10654-025-01346-2)
Supplement: Supplementary file 1 — Supplementary Material 1 [file 10654_2025_1346_MOESM1_ESM.docx]

Supplementary Material 1

Table S1. Sample size by country and sex applied in the estimations based on the Sullivan and HCAL methods.

|  | Women | | | | Men | | | |
| --- | --- | --- | --- | --- | --- | --- | --- | --- |
| Country | Chronic Diseases | Self-rated Health | Functional Limitations | Disability | Chronic Diseases | Self-rated Health | Functional Limitations | Disability |
| AUT | 1841 | 1846 | 1846 | 1835 | 1259 | 1264 | 1264 | 1256 |
| BEL | 2698 | 2699 | 2699 | 2681 | 2127 | 2130 | 2130 | 2114 |
| BGR | 1126 | 1130 | 1130 | 1118 | 817 | 818 | 818 | 812 |
| CHE | 1297 | 1298 | 1299 | 1290 | 1056 | 1057 | 1057 | 1049 |
| CZE | 2454 | 2458 | 2458 | 2437 | 1640 | 1642 | 1642 | 1631 |
| DEU | 1963 | 1964 | 1964 | 1922 | 1733 | 1738 | 1738 | 1708 |
| DNK | 1752 | 1754 | 1754 | 1741 | 1465 | 1466 | 1466 | 1460 |
| ESP | 2586 | 2591 | 2591 | 2580 | 2026 | 2029 | 2029 | 2021 |
| EST | 3109 | 3119 | 3119 | 3082 | 1946 | 1949 | 1949 | 1918 |
| FIN | 1064 | 1066 | 1066 | 1060 | 908 | 908 | 908 | 906 |
| FRA | 1881 | 1881 | 1881 | 1872 | 1343 | 1346 | 1345 | 1336 |
| GRC | 1763 | 1763 | 1763 | 1756 | 1290 | 1290 | 1290 | 1288 |
| HRV | 1325 | 1331 | 1331 | 1315 | 1057 | 1061 | 1061 | 1048 |
| HUN | 908 | 911 | 911 | 897 | 591 | 594 | 594 | 585 |
| ITA | 2501 | 2504 | 2504 | 2484 | 2023 | 2028 | 2028 | 2007 |
| LTU | 1280 | 1283 | 1283 | 1261 | 713 | 714 | 714 | 706 |
| LUX | 667 | 668 | 668 | 659 | 561 | 563 | 563 | 544 |
| LVA | 1081 | 1083 | 1083 | 1075 | 618 | 619 | 619 | 614 |
| POL | 707 | 708 | 708 | 703 | 548 | 550 | 550 | 543 |
| PRT | 2573 | 2577 | 2577 | 2558 | 2054 | 2060 | 2060 | 2041 |
| SVK | 1115 | 1122 | 1122 | 1104 | 939 | 950 | 950 | 935 |
| SVN | 2114 | 2117 | 2117 | 2102 | 1523 | 1525 | 1525 | 1512 |
| SWE | 1711 | 1712 | 1712 | 1705 | 1446 | 1450 | 1450 | 1446 |

Data Source: SHARE Wave 7.

Table S2. Sample size by country and sex applied in the estimations based on the multistate life tables method.

|  | Female | | | | Male | | | |
| --- | --- | --- | --- | --- | --- | --- | --- | --- |
| Country | Chronic Diseases | Self-rated Health | Functional Limitations | Disability | Chronic Diseases | Self-rated Health | Functional Limitations | Disability |
| AUT | 1515 | 1517 | 1517 | 1509 | 1032 | 1035 | 1035 | 1027 |
| BEL | 2397 | 2398 | 2398 | 2380 | 1932 | 1935 | 1935 | 1920 |
| CZE | 2208 | 2212 | 2212 | 2192 | 1459 | 1461 | 1461 | 1450 |
| DEU | 1807 | 1807 | 1807 | 1768 | 1639 | 1644 | 1644 | 1619 |
| DNK | 1592 | 1594 | 1594 | 1582 | 1354 | 1355 | 1355 | 1349 |
| ESP | 2255 | 2259 | 2259 | 2251 | 1799 | 1802 | 1802 | 1793 |
| EST | 2768 | 2777 | 2777 | 2745 | 1722 | 1725 | 1725 | 1700 |
| FRA | 1586 | 1586 | 1586 | 1579 | 1166 | 1169 | 1168 | 1160 |
| GRC | 1568 | 1568 | 1568 | 1561 | 1181 | 1181 | 1181 | 1180 |
| HRV | 1068 | 1071 | 1071 | 1057 | 865 | 867 | 867 | 855 |
| ITA | 2146 | 2148 | 2148 | 2129 | 1800 | 1805 | 1805 | 1786 |
| POL | 807 | 808 | 807 | 807 | 609 | 610 | 610 | 609 |
| SWE | 1598 | 1599 | 1599 | 1593 | 1355 | 1359 | 1359 | 1355 |

Data Source: SHARE Waves 6 and 7.

Table S3. Life expectancy* at age 50 by country and sex according to the estimations based on the multistate life tables method for the four health indicators.

|  | Female | | | | Male | | | |
| --- | --- | --- | --- | --- | --- | --- | --- | --- |
| Country | Chronic Diseases | Self-rated Health | Functional Limitations | Disability | Chronic Diseases | Self-rated Health | Functional Limitations | Disability |
| AUT | 36.0 | 36.1 | 35.5 | 36.5 | 30.6 | 30.6 | 31.2 | 30.9 |
| BEL | 33.6 | 33.5 | 33.9 | 34.2 | 30.4 | 28.5 | 30.6 | 30.6 |
| CZE | 32.9 | 34.6 | 33.4 | 33.1 | 26.5 | 30.0 | 28.0 | 27.6 |
| DEU | 35.6 | 35.4 | 37.3 | 36.2 | - | 28.9 | 29.3 | 28.3 |
| DNK | 32.2 | 30.9 | 31.3 | 31.7 | 30.0 | 28.8 | 29.5 | 30.3 |
| ESP | 33.7 | 33.6 | 33.0 | 33.1 | 28.8 | 28.2 | 27.6 | 28.6 |
| EST | 31.6 | 31.5 | 31.7 | 31.4 | 22.7 | 23.0 | 23.3 | 22.8 |
| FRA | 37.5 | 36.6 | 37.1 | 37.8 | 31.1 | 31.0 | 31.2 | 31.5 |
| GRC | 33.8 | 34.4 | 34.1 | 34.2 | 29.5 | 29.5 | 29.9 | 29.1 |
| HRV | 28.7 | 28.8 | 29.0 | 28.6 | 24.2 | 24.4 | 24.8 | 23.8 |
| ITA | 34.4 | 34.2 | 34.0 | 34.5 | 32.6 | 32.6 | 32.8 | 32.7 |
| POL | 29.5 | 29.6 | 29.0 | 29.5 | 24.1 | 24.3 | 23.7 | 23.9 |
| SWE | 35.6 | 35.5 | 35.9 | 35.6 | 33.1 | 32.5 | 32.9 | 33.4 |

Data Source: SHARE Waves 6 and 7.

*Note: See Appendix A2 below.

Table S4. Life expectancy (LE) and unhealthy life expectancy (ULY) at age 50 based on the Sullivan method, 2017, by country, sex, health indicator.

| Country | Women | | | | | Men | | | | |
| --- | --- | --- | --- | --- | --- | --- | --- | --- | --- | --- |
|  |  | ULY | | | |  | ULY | | | |
|  | LE | Chronic Diseases | Self-rated Health | Functional Limitations | Disability | LE | Chronic Diseases | Self-rated Health | Functional Limitations | Disability |
| AUT | 35 | 27.1 | 13.4 | 7.4 | 4.7 | 31 | 22.9 | 10.9 | 4.4 | 3 |
| BEL | 34.9 | 29 | 11.9 | 6.8 | 6.7 | 30.9 | 25 | 9.7 | 4.7 | 3.9 |
| BGR | 30.3 | 22.8 | 12.7 | 4.5 | 4.5 | 24.6 | 16.7 | 8 | 2.8 | 3 |
| CHE | 36.3 | 23.6 | 8.5 | 4.1 | 3.2 | 32.9 | 24.6 | 7.7 | 5 | 2.4 |
| CZE | 33 | 27.9 | 10.3 | 5.7 | 4.7 | 28 | 24.1 | 7.9 | 4.8 | 3.5 |
| DEU | 34.4 | 28.8 | 16.1 | 8.2 | 5.2 | 30.3 | 25.5 | 12.9 | 6.1 | 3.6 |
| DNK | 34.2 | 25.6 | 10.4 | 4.6 | 3.3 | 30.8 | 22.4 | 8.1 | 3.2 | 3 |
| ESP | 36.6 | 30 | 17.4 | 4.3 | 6.3 | 31.7 | 26.9 | 11.7 | 2 | 5.8 |
| EST | 33.6 | 27 | 23.6 | 9.5 | 5.2 | 26.6 | 20.2 | 18.1 | 7.2 | 3.2 |
| FIN | 35.4 | 30.3 | 14.5 | 4.4 | 3.7 | 30.8 | 26.4 | 13.1 | 4 | 2.6 |
| FRA | 36.6 | 30 | 14.5 | 6.4 | 5.3 | 31.5 | 24.5 | 11.9 | 5.3 | 3.9 |
| GRC | 34.8 | 26.5 | 12.3 | 2.9 | 3.1 | 30.6 | 21.3 | 8.1 | 1.7 | 1.7 |
| HRV | 32.1 | 26.6 | 15.7 | 6.4 | 4.9 | 27 | 20.9 | 11.3 | 4.9 | 2.9 |
| HUN | 30.7 | 24.6 | 18.3 | 6.5 | 3.1 | 24.8 | 16 | 11 | 3.6 | 1.9 |
| ITA | 35.9 | 26.4 | 17.6 | 6.4 | 5.5 | 32 | 22.1 | 12.3 | 4.4 | 2.8 |
| LTU | 32.3 | 28.1 | 20.7 | 5.4 | 5.5 | 24.8 | 19.8 | 13.7 | 4.1 | 3 |
| LUX | 34.9 | 28.8 | 14.9 | 7.3 | 3.6 | 31.3 | 25.5 | 11.7 | 6.6 | 3.5 |
| LVA | 31.5 | 26 | 21.6 | 4.5 | 4.2 | 24.1 | 17.6 | 14 | 3 | 1.9 |
| POL | 32.9 | 28.2 | 17.8 | 8.3 | 5.5 | 26.7 | 21.3 | 13.2 | 6 | 3.7 |
| PRT | 35.7 | 30.1 | 21.9 | 7.2 | 8.5 | 30.6 | 22.1 | 14 | 4.5 | 4.6 |
| SVK | 32 | 21.6 | 11.3 | 4.6 | 3.3 | 26.3 | 13.7 | 6.3 | 1.9 | 1.4 |
| SVN | 34.7 | 27.1 | 14.3 | 7.6 | 3.8 | 29.6 | 22.3 | 11.6 | 6.1 | 3.3 |
| WE | 35.1 | 25.4 | 11 | 6.1 | 3.4 | 32.4 | 22.5 | 8.4 | 4.3 | 2.8 |

Data Source: SHARE Wave 7 and Human Mortality Database (2024).

Table S5. Cross-sectional life expectancy (CAL) and cross-sectional unhealthy life expectancy (ULY) at age 50, 2017, by country, sex, health indicator.

|  | Women | | | | | Men | | | | |
| --- | --- | --- | --- | --- | --- | --- | --- | --- | --- | --- |
| Country | CAL | ULY | | | | CAL | ULY | | | |
|  |  | Chronic diseases | Self-rated Health | Functional Limitations | Disability |  | Chronic diseases | Self-rated Health | Functional Limitations | Disability |
| AUT | 34.8 | 27 | 13.3 | 7.4 | 4.6 | 30.8 | 22.7 | 10.8 | 4.4 | 3 |
| BEL | 34.8 | 28.9 | 11.9 | 6.8 | 6.6 | 30.8 | 24.8 | 9.6 | 4.6 | 3.9 |
| BGR | 30.2 | 22.7 | 12.6 | 4.4 | 4.5 | 24.4 | 16.6 | 8 | 2.8 | 2.9 |
| CHE | 36.2 | 23.5 | 8.4 | 4.1 | 3.1 | 32.8 | 24.6 | 7.6 | 5 | 2.4 |
| CZE | 32.9 | 27.8 | 10.2 | 5.7 | 4.7 | 27.9 | 24 | 7.8 | 4.7 | 3.4 |
| DEU | 34.4 | 28.8 | 16.1 | 8.2 | 5.2 | 30.2 | 25.4 | 12.9 | 6.1 | 3.6 |
| DNK | 34 | 25.5 | 10.4 | 4.5 | 3.3 | 30.6 | 22.3 | 8.1 | 3.2 | 3 |
| ESP | 36.7 | 30.1 | 17.4 | 4.3 | 6.4 | 31.6 | 26.8 | 11.7 | 2 | 5.8 |
| EST | 33.6 | 26.9 | 23.6 | 9.5 | 5.2 | 26.3 | 20 | 17.8 | 7 | 3.1 |
| FIN | 35.3 | 30.2 | 14.5 | 4.4 | 3.7 | 30.7 | 26.3 | 13 | 4 | 2.6 |
| FRA | 36.6 | 29.9 | 14.5 | 6.4 | 5.3 | 31.4 | 24.4 | 11.8 | 5.3 | 3.9 |
| GRC | 34.9 | 26.6 | 12.4 | 2.9 | 3.1 | 30.7 | 21.4 | 8.2 | 1.7 | 1.7 |
| HUN | 30.6 | 24.6 | 18.3 | 6.4 | 3.1 | 24.7 | 15.9 | 10.9 | 3.6 | 1.9 |
| ITA | 35.9 | 26.4 | 17.6 | 6.5 | 5.5 | 32 | 22.1 | 12.3 | 4.4 | 2.8 |
| LTU | 32.2 | 28 | 20.6 | 5.4 | 5.5 | 24.4 | 19.4 | 13.5 | 4 | 2.9 |
| LUX | 35.3 | 29.2 | 15.1 | 7.4 | 3.7 | 31.2 | 25.4 | 11.7 | 6.6 | 3.4 |
| LVA | 31.5 | 26 | 21.6 | 4.5 | 4.2 | 24 | 17.5 | 13.9 | 3 | 1.9 |
| POL | 32.8 | 28.2 | 17.7 | 8.3 | 5.5 | 26.6 | 21.2 | 13.2 | 6 | 3.7 |
| PRT | 35.5 | 30 | 21.8 | 7.1 | 8.4 | 30.3 | 21.9 | 13.9 | 4.4 | 4.6 |
| SVK | 31.9 | 21.5 | 11.2 | 4.5 | 3.3 | 26.3 | 13.7 | 6.2 | 1.9 | 1.4 |
| SVN | 34.6 | 27 | 14.3 | 7.6 | 3.8 | 29.6 | 22.2 | 11.6 | 6.1 | 3.3 |
| SWE | 35.1 | 25.4 | 11 | 6.1 | 3.4 | 32.2 | 22.4 | 8.4 | 4.3 | 2.7 |

Data Source: SHARE Wave 7 and Human Mortality Database (2024).

Table S6: Life expectancy (LE), health expectancy (HLE), and unhealthy life expectancy (ULY) at age 50 based on the multistate life tables method. 2015-2017 , by country, sex, health indicator.

| Country | Women | | | | | Men | | | | |
| --- | --- | --- | --- | --- | --- | --- | --- | --- | --- | --- |
|  | LE | ULY | | | | LE | ULY | | | |
|  |  | Chronic Diseases | Self-rated Health | Functional Limitations | Disability |  | Chronic Diseases | Self-rated Health | Functional Limitations | Disability |
| AUT | 35 | 27 | 12.4 | 7.7 | 3.8 | 31 | 21.9 | 10.1 | 4.3 | 2.6 |
| BEL | 34.9 | 28.7 | 12.5 | 6.2 | 5.8 | 30.9 | 25.8 | 12.5 | 4.4 | 3.8 |
| CZE | 33 | 28.4 | 7.3 | 4.9 | 4 | 28 | 24.5 | 5.2 | 3.8 | 3.3 |
| DEU | 34.4 | 28.8 | 16.2 | 7.5 | 4.2 | 30.3 | - | 14.4 | 6.1 | 3.8 |
| DNK | 34.2 | 24.7 | 11.2 | 4.2 | 2.8 | 30.8 | 22.9 | 8.7 | 3.2 | 2.8 |
| ESP | 36.6 | 29.7 | 16.8 | 4.7 | 6.6 | 31.7 | 24.8 | 12.6 | 2.3 | 2.6 |
| EST | 33.6 | 26.4 | 23.9 | 9 | 5.1 | 26.6 | 20.8 | 18.1 | 6.3 | 3.3 |
| FRA | 36.6 | 29.6 | 13.9 | 4.8 | 3.8 | 31.5 | 24.3 | 11.3 | 4.1 | 2.5 |
| GRC | 34.8 | 24.9 | 10.3 | 2.4 | 2.4 | 30.6 | 19.1 | 7.3 | 1.1 | 1.4 |
| HRV | 32.1 | 26.2 | 15.3 | 6.1 | 4.5 | 27 | 22 | 11.4 | 4.4 | 2.9 |
| ITA | 35.9 | 26.1 | 17.1 | 6.5 | 4.7 | 32 | 20.7 | 12.6 | 4.3 | 2.3 |
| POL | 32.9 | 26.6 | 16.5 | 7.5 | 4.9 | 26.7 | 21.4 | 13.9 | 6.9 | 3.6 |
| SWE | 35.1 | 26 | 10.4 | 5.8 | 2.6 | 32.4 | 24.2 | 9.3 | 4 | 2.6 |

Notes: No values for Germany for chronic diseases since there was only one transition from healthy to death between the waves.

Data Source: SHARE Waves 6 and 7 and Human Mortality Database (2024).

Table S7. Gender gap (women - men) in unhealthy life expectancy at age 50, the contribution of the effect of gender differences in mortality and health in the Sullivan method, 2017, by country, health indicator.

|  |  | Disability | | Chronic Diseases | | | Functional Limitations | | | Self-rated Health | | |
| --- | --- | --- | --- | --- | --- | --- | --- | --- | --- | --- | --- | --- |
| Country | Gap | Mortality | Health | Gap | Mortality | Health | Gap | Mortality | Health | Gap | Mortality | Health |
| AUT | 1.6 | 1.0 | 0.6 | 4.2 | 3.4 | 0.8 | 3.0 | 1.2 | 1.8 | 2.5 | 2.0 | 0.5 |
| BEL | 2.7 | 1.2 | 1.6 | 4.0 | 3.5 | 0.4 | 2.1 | 1.0 | 1.1 | 2.2 | 1.5 | 0.7 |
| BGR | 1.6 | 1.4 | 0.1 | 6.1 | 4.9 | 1.2 | 1.6 | 1.3 | 0.3 | 4.6 | 3.1 | 1.5 |
| CHE | 0.8 | 0.5 | 0.3 | -1.0 | 2.8 | -3.8 | -0.9 | 0.5 | -1.4 | 0.8 | 1.1 | -0.2 |
| CZE | 1.3 | 1.2 | 0.1 | 3.8 | 4.5 | -0.7 | 0.9 | 1.3 | -0.4 | 2.4 | 2.0 | 0.4 |
| DEU | 1.6 | 1.1 | 0.5 | 3.3 | 3.7 | -0.4 | 2.1 | 1.3 | 0.8 | 3.2 | 2.3 | 0.9 |
| DNK | 0.3 | 0.7 | -0.4 | 3.2 | 2.8 | 0.4 | 1.4 | 0.7 | 0.7 | 2.3 | 1.4 | 0.9 |
| ESP | 0.6 | 1.6 | -1.0 | 3.2 | 4.5 | -1.4 | 2.2 | 0.8 | 1.4 | 5.7 | 3.0 | 2.6 |
| EST | 2.0 | 1.7 | 0.3 | 6.7 | 6.2 | 0.6 | 2.4 | 2.8 | -0.4 | 5.5 | 5.7 | -0.2 |
| FIN | 1.1 | 0.8 | 0.3 | 3.9 | 4.2 | -0.3 | 0.4 | 1.1 | -0.7 | 1.4 | 2.7 | -1.3 |
| FRA | 1.4 | 1.4 | 0.1 | 5.4 | 4.5 | 0.9 | 1.1 | 1.6 | -0.5 | 2.6 | 2.6 | 0.0 |
| GRC | 1.4 | 0.6 | 0.8 | 5.2 | 3.8 | 1.4 | 1.2 | 0.5 | 0.7 | 4.2 | 2.2 | 1.9 |
| HRV | 1.9 | 1.2 | 0.7 | 5.7 | 4.5 | 1.2 | 1.6 | 1.4 | 0.2 | 4.4 | 2.8 | 1.6 |
| HUN | 1.2 | 1.1 | 0.1 | 8.7 | 5.2 | 3.5 | 2.8 | 1.6 | 1.2 | 7.4 | 4.0 | 3.3 |
| ITA | 2.7 | 1.2 | 1.5 | 4.2 | 3.4 | 0.8 | 2.0 | 1.2 | 0.8 | 5.3 | 2.4 | 2.8 |
| LTU | 2.5 | 1.9 | 0.6 | 8.4 | 6.6 | 1.7 | 1.3 | 1.9 | -0.5 | 7.0 | 5.3 | 1.7 |
| LUX | 0.2 | 0.9 | -0.7 | 3.3 | 3.1 | 0.1 | 0.7 | 1.2 | -0.4 | 3.2 | 1.8 | 1.4 |
| LVA | 2.3 | 1.3 | 1.0 | 8.4 | 6.5 | 2.0 | 1.5 | 1.4 | 0.1 | 7.7 | 5.7 | 2.0 |
| POL | 1.8 | 1.6 | 0.2 | 6.9 | 5.6 | 1.4 | 2.3 | 2.2 | 0.1 | 4.5 | 4.1 | 0.5 |
| PRT | 3.8 | 1.5 | 2.3 | 8.0 | 4.6 | 3.5 | 2.7 | 1.5 | 1.2 | 7.9 | 3.2 | 4.7 |
| SVK | 1.9 | 0.8 | 1.1 | 7.9 | 4.2 | 3.7 | 2.6 | 1.0 | 1.7 | 5.0 | 2.7 | 2.3 |
| SVN | 0.5 | 1.1 | -0.6 | 4.8 | 4.5 | 0.3 | 1.5 | 1.7 | -0.2 | 2.7 | 2.9 | -0.2 |
| SWE | 0.6 | 0.6 | 0.1 | 2.9 | 2.4 | 0.6 | 1.8 | 0.7 | 1.1 | 2.6 | 1.2 | 1.4 |

Data Source: SHARE Wave 7 and Human Mortality Database (2024).

Table S8. Gender gap (women - men) in cross-sectional unhealthy life expectancy at age 50, the contribution of gender differences in mortality and morbidity, 2017, by country, health indicator.

|  | Chronic Diseases | | | Self-rated Health | | | Functional Limitations | | |  | Disability | |
| --- | --- | --- | --- | --- | --- | --- | --- | --- | --- | --- | --- | --- |
| Country | Gap | Mortality | Health | Gap | Mortality | Health | Gap | Mortality | Health | Gap | Mortality | Health |
| AUT | 4.2 | 3.4 | 0.8 | 2.5 | 2 | 0.5 | 3 | 1.2 | 1.8 | 1.6 | 1 | 0.6 |
| BEL | 4 | 3.5 | 0.4 | 2.2 | 1.5 | 0.7 | 2.1 | 1 | 1.1 | 2.7 | 1.2 | 1.6 |
| BGR | 6.1 | 4.9 | 1.2 | 4.6 | 3.1 | 1.5 | 1.6 | 1.3 | 0.3 | 1.6 | 1.4 | 0.1 |
| CHE | -1 | 2.8 | -3.8 | 0.8 | 1.1 | -0.2 | -0.9 | 0.5 | -1.4 | 0.8 | 0.5 | 0.3 |
| CZE | 3.8 | 4.5 | -0.7 | 2.4 | 2 | 0.4 | 0.9 | 1.3 | -0.4 | 1.3 | 1.2 | 0.1 |
| DEU | 3.3 | 3.7 | -0.4 | 3.2 | 2.3 | 0.9 | 2.1 | 1.3 | 0.8 | 1.6 | 1.1 | 0.5 |
| DNK | 3.2 | 2.8 | 0.4 | 2.3 | 1.4 | 0.9 | 1.4 | 0.7 | 0.7 | 0.3 | 0.7 | -0.4 |
| ESP | 3.2 | 4.5 | -1.4 | 5.7 | 3 | 2.6 | 2.2 | 0.8 | 1.4 | 0.6 | 1.6 | -1 |
| EST | 6.7 | 6.2 | 0.6 | 5.5 | 5.7 | -0.2 | 2.4 | 2.8 | -0.4 | 2 | 1.7 | 0.3 |
| FIN | 3.9 | 4.2 | -0.3 | 1.4 | 2.7 | -1.3 | 0.4 | 1.1 | -0.7 | 1.1 | 0.8 | 0.3 |
| FRA | 5.4 | 4.5 | 0.9 | 2.6 | 2.6 | 0 | 1.1 | 1.6 | -0.5 | 1.4 | 1.4 | 0.1 |
| GRC | 5.2 | 3.8 | 1.4 | 4.2 | 2.2 | 1.9 | 1.2 | 0.5 | 0.7 | 1.4 | 0.6 | 0.8 |
| HRV | 5.7 | 4.5 | 1.2 | 4.4 | 2.8 | 1.6 | 1.6 | 1.4 | 0.2 | 1.9 | 1.2 | 0.7 |
| HUN | 8.7 | 5.2 | 3.5 | 7.4 | 4 | 3.3 | 2.8 | 1.6 | 1.2 | 1.2 | 1.1 | 0.1 |
| ITA | 4.2 | 3.4 | 0.8 | 5.3 | 2.4 | 2.8 | 2 | 1.2 | 0.8 | 2.7 | 1.2 | 1.5 |
| LTU | 8.4 | 6.6 | 1.7 | 7 | 5.3 | 1.7 | 1.3 | 1.9 | -0.5 | 2.5 | 1.9 | 0.6 |
| LUX | 3.3 | 3.1 | 0.1 | 3.2 | 1.8 | 1.4 | 0.7 | 1.2 | -0.4 | 0.2 | 0.9 | -0.7 |
| LVA | 8.4 | 6.5 | 2 | 7.7 | 5.7 | 2 | 1.5 | 1.4 | 0.1 | 2.3 | 1.3 | 1 |
| POL | 6.9 | 5.6 | 1.4 | 4.5 | 4.1 | 0.5 | 2.3 | 2.2 | 0.1 | 1.8 | 1.6 | 0.2 |
| PRT | 8 | 4.6 | 3.5 | 7.9 | 3.2 | 4.7 | 2.7 | 1.5 | 1.2 | 3.8 | 1.5 | 2.3 |
| SVK | 7.9 | 4.2 | 3.7 | 5 | 2.7 | 2.3 | 2.6 | 1 | 1.7 | 1.9 | 0.8 | 1.1 |
| SVN | 4.8 | 4.5 | 0.3 | 2.7 | 2.9 | -0.2 | 1.5 | 1.7 | -0.2 | 0.5 | 1.1 | -0.6 |
| SWE | 2.9 | 2.4 | 0.6 | 2.6 | 1.2 | 1.4 | 1.8 | 0.7 | 1.1 | 0.6 | 0.6 | 0.1 |

Data Source: SHARE Wave 7 and Human Mortality Database (2024).

Table S9. Gender gap (women - men) in unhealthy life expectancy at age 50, the contribution of gender differences in mortality and morbidity based on the multistate life table method. 2017.

|  | Chronic Diseases | | | Self-rated Health | | | Functional Limitations | | |  | Disability |  |
| --- | --- | --- | --- | --- | --- | --- | --- | --- | --- | --- | --- | --- |
| Country | Gap | Mortality | Health | Gap | Mortality | Health | Gap | Mortality | Health | Gap | Mortality | Health |
| AUT | 5.1 | 3.4 | 1.6 | 2.4 | 1.9 | 0.5 | 3.3 | 1 | 2.3 | 1.2 | 0.9 | 0.3 |
| BEL | 2.9 | 3.6 | -0.6 | 0.1 | 1.5 | -1.4 | 1.8 | 0.8 | 1 | 2 | 1 | 1 |
| CZE | 3.9 | 4.5 | -0.7 | 2.1 | 1.4 | 0.6 | 1.2 | 1.1 | 0 | 0.7 | 1.1 | -0.3 |
| DEU | - | - | - | 1.7 | 2.3 | -0.5 | 1.4 | 1.1 | 0.3 | 0.4 | 0.9 | -0.5 |
| DNK | 1.9 | 2.9 | -1.1 | 2.5 | 1.3 | 1.2 | 1 | 0.6 | 0.3 | 0.1 | 0.5 | -0.4 |
| ESP | 4.9 | 4.5 | 0.4 | 4.2 | 3 | 1.2 | 2.3 | 0.7 | 1.6 | 4 | 1.4 | 2.7 |
| EST | 5.6 | 6.2 | -0.7 | 5.8 | 5.8 | 0 | 2.8 | 2.6 | 0.2 | 1.8 | 1.7 | 0.1 |
| FRA | 5.3 | 4.4 | 0.8 | 2.6 | 2.3 | 0.3 | 0.7 | 1.1 | -0.4 | 1.3 | 0.9 | 0.4 |
| GRC | 5.8 | 3.7 | 2.1 | 3 | 2.1 | 0.9 | 1.3 | 0.5 | 0.8 | 1 | 0.5 | 0.5 |
| HRV | 4.2 | 4.5 | -0.3 | 4 | 2.7 | 1.2 | 1.7 | 1.2 | 0.4 | 1.6 | 1.2 | 0.4 |
| ITA | 5.3 | 3.4 | 1.9 | 4.5 | 2.5 | 2 | 2.2 | 1.1 | 1.1 | 2.4 | 0.9 | 1.5 |
| POL | 5.2 | 5.5 | -0.3 | 2.6 | 3.9 | -1.3 | 0.6 | 2.1 | -1.5 | 1.3 | 1.5 | -0.2 |
| SWE | 1.8 | 2.4 | -0.6 | 1.1 | 1.2 | -0.2 | 1.8 | 0.7 | 1.2 | 0 | 0.5 | -0.5 |

Notes: No values for Germany for chronic diseases since for men there was only one transition from healthy to death between the waves.

Data Source: SHARE Wave 6 and 7 and Human Mortality Database (2024).

Appendix

*A1 - Survey questions and categories*

For the prevalence, we focused on the four most frequently used self-assessed measures of health: (i) presence of chronic diseases, (ii) presence of functional limitations, (iii) poor self-rated health (SRH), and (iv) limitations in the activities of daily living (ADL). Chronic diseases were defined as the presence of longstanding health problems based on the question “Do you have any longstanding illness or health problems?” with the two possible answers “yes” and “no.” Functional limitations were measured with the “Global Activity Limitations Indicator” (GALI) which is based on the question “For at least the past six months, to what extent have you been limited because of a health problem in activities people usually do?” with the three possible responses “severely limited,” “limited, but not severely,” and “not limited at all.” SRH reflects the respondents’ subjective evaluation of their health based on the question “Would you say your health is...” with the response categories “excellent”, “very good”, “good”, “fair”, “poor”. Finally, disability in ADL was assessed through difficulty in dressing, bathing or showering, eating, walking across a room, getting in/out of a bed, and toileting. For each of these activities, respondents were asked: “Please tell me whether you have any difficulty doing each of the everyday activities on this card. Exclude any difficulties that you expect to last less than three months.” Poor health was defined on these four dimensions as follows: presence of chronic diseases (answer “yes”), or functional limitations (“limited, but not strongly” and “strongly limited” combined), poor SRH (“fair” and “poor” combined), or disability (inability to perform at least one of the six ADLs).

A2 - Three Methods to Estimate ULY

The average number of life years spent unhealthy starting at age $a$ ($a$=50 in our study, i.e., ULY at age 50) is derived as:

${ULY}_{a}=\frac{1}{l_{a}}\sum_{x=a}^{\omega} \pi_{x}L_{x} ,$ (1)

where $a$ is the life table starting age, $l_{a}$ is the number of persons of the life table population alive at age *a*, and *ω* is the maximum attainable age. $\pi_{x}$refers to the prevalence of a specific health state at age x, and $L_{x}$ is the number of years lived at age *x* by the life table population (because of data restrictions, in this study we used 5-year age groups, with the highest open ended age group being 90+).

The first method we used to estimate ULY is the Sullivan method, in which the prevalence of a specific health state at a given age is estimated from cross-sectional survey data. The distribution of life years lived by age is taken from a period life table, which summarizes the age-specific mortality rates observed during the calendar year(s) to which the life table refers.

Additionally, following an extension of the “cross-sectional average length of life” approach (CAL), we used HCAL as a second measure of ULY. HCAL stands for the cross-sectional average length of “healthy” life, i.e., CAL in a specific health state. As in the case of the Sullivan method, the health state prevalence ($\pi_{x}$) in HCAL is derived from cross-sectional survey data. The difference between the two approaches is the number of years lived at age *x* in a given period. Whereas the Sullivan approach uses life years from a period life table, HCAL applies years lived at age x during the study period, taken from the cohort life table of the cohort aged x at that time.

The third method is the multistate life table approach, in which the estimates of ULY are based on transition rates between health states during the study period, with death being the absorbing state. The probabilities of transition between health states are derived from the health states at selected points in time at individual level in a longitudinal survey. Based on these probabilities, we derive the prevalence of a specific health state by age. Instead of using the distribution of years lived derived directly from multistate models, as is commonly done, we rely on the distribution of years lived in the period life table population for the study period. The objective is to make the results meaningfully comparable across the various health dimensions of the study. As shown in Table S3 above, the number of years lived, as estimated by the multistate models, varies across models with different health dimensions.

In summary, the three variants of ULY measure different aspects of population health and mortality conditions in a study period. ULY derived with the Sullivan method summarizes age-specific mortality rates and prevalence of the defined state of adverse health condition in the life table population of a study period. ULY based on the HCAL approach measures the average number of unhealthy years lived in a given period over all cohorts alive in that period. ULY derived with the multistate life tables summarizes the outcome of the health processes that occur in the study population during a given period. The Sullivan method has the least data requirements. It is simple and widely used, but it was questioned as an appropriate measure of population health in the situation of rapid changes in health transitions or dynamic population health shifts. The HCAL approach aims for a better conceptual fit between mortality and health data by incorporating cohort mortality histories. It is based on the understanding that health prevalence observed in a study period results not only from the health conditions during the observation year(s), but also from the health conditions experienced by the cohorts over their complete lifetime from birth until the study period. The data to construct cohort life tables for the estimation of CAL was available for all countries in this study, with Croatia being the only exception. Multistate models allow for dynamic modeling of health transitions, which gives a more accurate picture of period-specific population health when health conditions change rapidly over time. However, the data required to estimate transition probabilities between selected health states and was available only for a subset of 13 countries (see Table S2 above).
